# Mental Health Conditions, Including Depression and Stress, Are Associated with Increased Odds of Gastric Cancer—Insights into the Role of Diet: A Case-Control Study

**DOI:** 10.3390/nu15234981

**Published:** 2023-11-30

**Authors:** Farhad Vahid, Wena Rahmani, Sayed Hossein Davoodi, Torsten Bohn

**Affiliations:** 1 Nutrition and Health Research Group, Department of Precision Health, Luxembourg Institute of Health, 1445 Strassen, Luxembourg; farhad.vahid@lih.lu; 2 School of Medicine, Arak University of Medical Science, Arak 3848176941, Iran; 3 Department of Cellular and Molecular Nutrition, Faculty of Nutrition and Food Technology, Shahid Beheshti University of Medical Sciences, Tehran 1981619573, Iran

**Keywords:** vitamin D, beta-carotene, inflammation, oxidative stress, pyridoxine

## Abstract

Several risk factors, including nutritional/lifestyle ones, play a role in gastric cancer etiology. Further interactions with mental health have also been emphasized. We hypothesized that individuals with mental disorders would exhibit compromised nutrient intake, increasing their risk of gastric cancer. The state of mental health was evaluated in 82 patients with gastric cancer and 95 healthy controls using the 21-item Depression–Anxiety–Stress Scale. The participants’ dietary intakes were evaluated by a 168-item food frequency questionnaire. Based on fully adjusted logistic regressions, there was a significant association between depression (OR = 1.938, CI 95%: 1.009–3.723) and stress (OR = 2.630, CI 95%: 1.014–6.819) with increased odds of gastric cancer. According to fully adjusted multinomial regressions, vitamins A and B6, beta-carotene, and black tea decreased the odds of depression, based on comparing the control group with cases of depression, while sugar and salt increased its odds. The highest significant association was found for salt intake and anxiety in cases with present anxiety (OR = 4.899, 95% CI: 2.218–10.819), and the highest significant protective effect was found for vitamin B6 and depression in cases with present depression (OR = 0.132, 95% CI: 0.055–0.320). However, considering causal relationships and clarifying the underlying mechanisms is imperative and requires further investigation. Advising healthy dietary patterns, e.g., a Mediterranean diet rich in vitamins, minerals, and phytochemicals such as vitamin A, B6, beta-carotene, and fiber, is expected to reduce the odds of gastric cancer, possibly related to lower levels of anxiety and depression.

## 1. Introduction

Cancer, as a global public health concern, has always been one of the main health threats. One of the cancers with the highest mortality rate and prevalence is gastric cancer (the fifth most common cancer worldwide) [1], with about one million new cases and more than 800 thousand deaths from gastric cancer being reported worldwide per annum [1]. Iran was among the 10 countries with the highest rates of gastric cancer and the highest number of deaths from gastric cancer in 2020. Iran ranks ninth in terms of the number of gastric cancer deaths, with age-standardized rates (ASR)/100,000 of 17.5, and sixth in gastric cancer incidence, with 15.5 ASR/100,000 worldwide [1].

Several factors and contributing risks have been reported to be associated with the risk of the incidence of gastric cancer as a heterogeneous disease. There is evidence that smoking, *Helicobacter pylori* (*H. pylori*) infection, and exposure to industrial chemicals, such as asbestos, increase the risk of gastric cancer [2,3,4]. There is also strong evidence that salt-preserved foods, high salt intake, alcoholic drinks, and being overweight or obese increase the risk of gastric cancer [1,5]. Some evidence also shows that consuming grilled or barbecued meat/fish and processed meat and little or no fruits are related to increased risk of gastric cancer [1,5]. In addition, low intake/deficiency of some micronutrients, namely vitamins D, A, and C, has been shown to be associated with some types of gastric cancer [6,7,8]. For instance, studies have shown that vitamin D deficiency is widespread in patients with cancer, including gastric cancer (87.3% according to the reference value of 20 ng/mL in serum) [9]. However, the epidemiological data remain inconsistent, as some retrospective observational studies have demonstrated the benefits of vitamin D supplementation, while a limited number of randomized controlled clinical trials have failed to clearly support the beneficial role of vitamin D supplementation in gastric cancers [6,7]. In addition, the beneficial effects of certain bioactive compounds, such as secondary plant metabolites, e.g., antioxidants such as beta-carotene and curcumin, and their protective effect on gastric cancer have been emphasized, though findings remain inconclusive [10,11,12].

As one of the main risk factors includes nutritional aspects, one of the factors that can affect eating habits/dietary patterns and food choices is a person’s mental and psychological state [13]. Studies have shown that psychological stress, depression, and anxiety can affect appetite, food choices, eating habits/patterns, and vice versa [13,14,15]. For instance, psychological stress-induced eating might be one factor contributing to the development of obesity [14]. However, stress appears to constitute a double-edged sword, resulting in either under- or overeating, which may be influenced by stressor severity [14]. However, investigating biological markers of stress will assist our understanding of the physiological mechanism underlying the stress–eating relationship and how stress might be linked to neurotransmitters and hormones that control appetite, food choices, and eating habits/patterns. Some studies have investigated the possibility of using dietary interventions to treat stress, anxiety, and depression [16], and demonstrated that dietary interventions, i.e., supplementation of multiple micronutrients, fish oils and intake of plant-based foods, resulted in beneficial effects and reduced odds of stress, depression, and anxiety [16].

On the other hand, studies have investigated the prevalence of stress, depression, and anxiety in patients with gastric cancer and reported that, e.g., 15.9% of patients had mild anxiety, 32% symptomatic anxiety, and mild and symptomatic depression were observed in 25% and 28% of patients, respectively [17]. Another study in Iran reported a high prevalence of these disorders. Overall, 57% and 47% of patients with gastrointestinal cancer scored high for depression and anxiety, respectively [18].

Although the prevalence of these disorders appears high, to the best of our knowledge, no study has investigated the triangular association between diet, mental health, and the risk of gastric cancer. Therefore, we have designed this study to investigate the association between psychological stress, depression, and anxiety and the odds of gastric cancer, considering dietary intake. We hypothesized that psychological stress, depression, and anxiety, either directly or through dietary intake, are related to increased odds of gastric cancer.

## 2. Materials and Methods

### 2.1. Participants

The full protocol and methodology of the study have been published elsewhere [19]. Briefly, this hospital-based case–control study was accomplished at a specialized clinic in the northwest of Iran (from 2014 to 2016). The survey included 82 patients with gastric cancer and 95 healthy controls. In the month prior to the study, a gastroenterologist diagnosed the (new) cases with gastric cancer. In addition, controls were selected randomly from other patients’ caregivers who were attending the same clinic. Cases and controls were frequency matched by age (±5 years) and sex. Informed consent was obtained from each participant after they had received written and verbal explanations about the study’s methodology/protocol. The study design and protocol complied with the ethical principles laid out in the 2008 revision of the Declaration of Helsinki and was approved by the Ethics Review Committee at Shahid Beheshti University of Medical Sciences, Tehran, Iran, prior to the study launch.

### 2.2. Inclusion and Exclusion Criteria

The inclusion criteria were the following: (a) not having any malignancy (except for gastric cancer in cases), (b) not having any conditions such as lactation, pregnancy, or a history of hepatic, neurological, gastrointestinal, renal, endocrine, immunological, or CVD issues, (c) not following any particular dietary patterns such as raw foodism/veganism or vegetarian, or any diet resulting in weight changes during the year prior to the interview, (d) to be in the age range of 20–80 years, and (e) willingness to participate in the study.

Exclusion criteria included the following: (a) major dietary changes during the study, (b) not sticking to the study protocol, and (c) reported daily energy intake outside the range of 800–6500 kcal (±3 SD of participants’ mean).

### 2.3. Assessment of Depression–Anxiety–Stress (DASS-21) Status

In this research, the state of symptoms of depression, anxiety, and stress was evaluated by completing the 21-item short form of the Depression–Anxiety–Stress Scale-21 (DASS-21) questionnaire. This valid and reliable questionnaire [20,21,22], designed by Lovibond and Lovibond [23], comprises a set of three self-report scales used to assess the negative emotional states of depression, anxiety, and stress used in various populations. To complete the questionnaire, a person needed to specify the status of symptoms. In this study, we asked the case group to specify the status of symptoms before diagnosis and the control group to specify their general and usual status. Each subscale of the DASS-21 included 7 questions, and the final score of each subscale was obtained by summing up the scores of the related questions. Each question was scored from 0 (not at all: does not apply to me at all) to 3 (very much: applies to me completely). Since DASS-21 is a shortened form of the main questionnaire (DASS-42), the final score of each of these subscales should be doubled, and then the severity of symptoms should be determined by referring to Table 1 [23]. It is worth mentioning that the scores of this questionnaire can be reported quantitatively without classification. However, in some of our analyses, we divided them into two groups: normal (stress ≤ 14, anxiety ≤ 7, and depression ≤ 9 points) and disordered (stress > 14, anxiety > 7, and depression > 9 points) (Table 1).

### 2.4. Assessment of Dietary Intake

In this study, the participants’ dietary intakes over the past year were evaluated by means of a 168-item quantitative, valid, and reliable food frequency questionnaire (FFQ) [24]. This FFQ inquired about the average intake (serving size) and frequency of consumption of 168 food items to provide a retrospective estimation of diet and mean time before cancer diagnosis. Individuals were asked to describe the frequency of each food item of FFQ in the last year according to the standard serving size. Depending on the type of food items, participants indicated their intake per day, week, month or year, or as never. Then, the data obtained from the FFQ were analyzed using Nutritionist version IV software for Windows (First Databank, Hearst Corp., San Bruno, CA, USA), and the average daily intake of nutrients and energy was calculated.

### 2.5. Assessment of Confounders and Other Baseline Variables

For all participants, information about gender (male, female), age (year), smoking (yes/no), education (≤high school diploma, >high school diploma), residency (urban, rural), H. pylori infection (positive, negative), cancer history in immediate family members (yes/no), regular physical activity (yes/no), and alcohol intake (yes/no) were collected through a general information questionnaire. The participants’ weight was recorded using a SECA digital scale with a 10 g accuracy, with persons dressed in light clothing. The height with an accuracy of 0.5 cm was measured without shoes in an upright position, leaning against the wall and shoulder blades under normal circumstances by a tape. Body mass index (BMI) was estimated by dividing weight (in kilograms) by the square height (in square meters). During several training sessions, the principal investigators trained a nutritionist, unaware of the study objectives, about completing the general information questionnaire and FFQ and doing the anthropometric measurements.

### 2.6. Assessment of Inflammatory and Oxidative Stress Biomarkers and Blood Samples

The participants were asked not to take any corticosteroids, anti-inflammatory medications, or painkillers at least 48 h before the blood draw. After fasting for 10–12 h, 10 mL of venous blood samples were taken (between 08:30–10:30 a.m.) in vacutainer tubes under sterile conditions. Serum was obtained from freshly drawn samples and was frozen at −70 °C until it was processed. The serum levels of inflammatory markers, including high-sensitivity C-reactive protein (hs-CRP) (mg/L), tumor necrosis factor-alpha (TNF-α) (pg/mL), interleukin-6 (IL-6, pg/mL), IL-1β (pg/mL), IL-4 (pg/mL), and IL-10 (pg/mL), along with fasting blood glucose (FBG, mg/dL) were measured. In addition, the serum levels of antioxidant biomarkers, including malondialdehyde (MDA) and total antioxidant capacity (TAC), were measured using thiobarbituric acid assay (TBA) and ferric-reducing antioxidant power (FRAP) methods. The inflammatory and antioxidant levels and FBG were measured using kits produced by Shanghai Crystal Day Biotech Co., Ltd. (Shanghai, China) and Pishtaz Teb Zaman Diagnostics Co., Ltd. (Tehran, Iran).

### 2.7. Data Treatment and Statistical Analysis

This study used IBM SPSS software (version 25) for statistical data analysis. Before choosing a statistical test, normality and equality of variances were investigated for continuous variables using the Kolmogorov–Smirnov (KS) test and Q-Q plots. Chi-square and Fisher’s exact test compared categorical variables between groups. The independent Student’s samples *t*-test was used to compare variables with normal distribution between groups. Variables that did not have a normal distribution were log-transformed. Pearson correlation was applied to investigate the correlation between dietary intakes (macro- and micronutrients) and scores of depression, anxiety, and stress in cases, controls, and the total sample.

Crude and multivariable-adjusted logistic and multinomial regression models were used to estimate odds ratios (ORs) and 95% confidence intervals (CIs). Multinomial logistic regression investigated the association between dietary intakes (macro- and micronutrients) as continuous variables and subgroups (four groups) of depression, anxiety, and stress. The logistic regression was applied to investigate the association between gastric cancer (case and controls) and depression, anxiety, and stress as continuous variables. Adjustments were made for age, gender, total energy intake, BMI, regular physical activity, smoking, cancer history in immediate family members, marital and education status, *H. pylori* infection, aspirin or NSAID use, and alcohol consumption in the adjusted models. Benjamini–Hochberg correction was applied to multicomparison analyses, and their *p*-values are reported after this correction. A *p*-value level below 0.05 was considered significant for all analyses.

## 3. Results

### 3.1. Baseline Characteristics

Significant differences were observed between cases and controls regarding regular physical activity and *H. pylori* infection (Table 2).

### 3.2. Comparison of Dietary Intakes in Cases and Controls across DASS-21 Subgroups

#### 3.2.1. Depression

**Figure 1:**
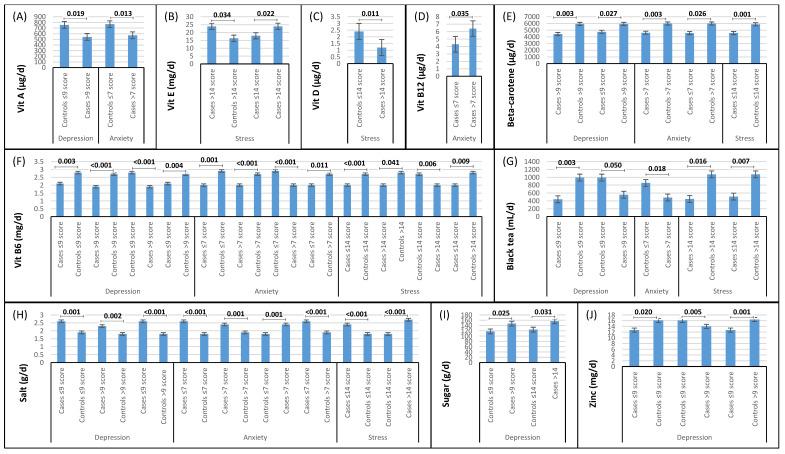
Comparison (one-way ANOVA with Tukey’s post hoc test) of dietary intakes (**A**) vitamin A, (**B**) vitamin E, (**C**) vitamin D, (**D**) vitamin B12, (**E**) beta-carotene, (**F**) vitamin B6, (**G**) black tea, (**H**) salt, (**I**) sugar, (**J**) zinc in cases and controls across depression, anxiety, and stress subgroups. Only significant *p*-values comparing two subgroups are shown; more details and non-significant values are given in Supplementary Tables S1–S3.

According to Figure 1 and Supplementary Table S1, controls with scores ≤ 9 for depression (normal), compared with cases with scores ≤ 9, had a significantly higher intake of vitamin B6, zinc, and black tea and consumed less salt. In addition, controls with scores > 9 for depression (disordered), compared with cases with scores > 9, exhibited a significantly higher intake of beta-carotene and vitamin B6 and consumed less salt. Comparing controls with and without depression with each other and cases with and without depression with each other did not show any significant different findings in dietary intake. Controls with scores ≤ 9 for depression (normal) compared with cases with scores > 9 (disordered) consumed significantly more vitamins A, B6, zinc, black tea, and less sugar and salt. In addition, cases with scores ≤ 9 for depression (normal) consumed significantly more fat and salt and less beta-carotene and vitamin B6 than controls with scores > 9 (disordered) (Figure 1 and Supplementary Table S1).

#### 3.2.2. Anxiety

Similar to results for the depression subgroups, controls with scores ≤ 7 for anxiety (normal) and controls with scores > 7 (disordered) compared with cases with scores ≤ 7 (normal) and cases with scores > 7 (disordered) had a significantly higher intake of vitamin B6 and consumed less salt (Figure 1 and Supplementary Table S2). Again, similar to the results of depression, comparing controls with and without anxiety with each other and cases with and without anxiety with each other did not show any significant results in terms of dietary intake. Meanwhile, controls with scores ≤ 7 for anxiety (normal), compared with cases with scores > 7 (disordered), significantly consumed more vitamins A and B6, as well as black tea, and less salt. In addition, cases with scores ≤ 7 for anxiety consumed significantly more salt and less beta-carotene and vitamin B6 than controls with scores > 7 (Figure 1 and Supplementary Table S2).

#### 3.2.3. Stress

According to Figure 1 and Supplementary Table S3, controls with scores ≤ 14 for stress (normal), compared with cases with scores ≤ 14 (normal), consumed significantly more vitamin B6, beta-carotene, and less salt. In addition, controls with scores > 14 for stress compared with cases with scores > 14 consumed more vitamins B6, E, and black tea. In addition, controls with scores ≤ 14 for stress compared with cases with scores > 14 consumed more vitamins B6 and D and less SFA, sugar, and salt, while cases with scores ≤ 14 for stress significantly consumed more vitamin B6 and black tea than cases with scores > 14 (Figure 1 and Supplementary Table S3).

### 3.3. Comparison of Inflammatory and Oxidative Stress Biomarkers in Cases and Controls across DASS-21 Subgroups

The comparison of inflammatory and oxidative stress biomarkers and FBG showed that patients with gastric cancer had significantly higher values for all measurements, except for anti-inflammatory IL-10, which was significantly higher in the controls, and IL-4, which showed no significant difference (Table 3). In addition, the investigation of depression subgroups showed that comparing individuals with normal depression scores with the group with high scores resulted in significantly higher values of IL-6 and FBG and significantly lower values of IL-. Comparison of anxiety subgroups showed that individuals with normal anxiety scores had significantly lower MDA values. In addition, comparison of stress subgroups also showed that individuals with normal stress scores had significantly lower TNF-α values.

### 3.4. Correlation Analyses

Based on Supplementary Table S4, there was a significant (weak and mild) correlation between depression scores in cases with vitamins C, B6, B12, and zinc; and in controls with PUFA, zinc, and sugar, and in total simple with vitamins C and sugar; between anxiety scores in controls with vitamins A, B12, and niacin, in the total sample with vitamin A, and black tea; and between stress scores in cases with vitamin E, in controls with protein, beta-carotene, and black tea, and in the total sample with protein, sugar, and salt. In addition, Supplementary Table S4 shows the strongest inverse correlation between vitamin E and anxiety scores in cases (r = −0.352, *p*-value = 0.001), and the strongest positive correlation was found between sugar and depression scores in controls (r = 0.331, *p*-value = 0.001).

### 3.5. Regression Models

#### 3.5.1. Multinomial Logistic Regression Models

Fully adjusted multinomial logistic regressions between dietary intakes and subgroups of depression, anxiety, and stress are shown in Table 4. Considering controls under the threshold score of depression (normal) as the reference group, findings showed that the intake of MUFA and vitamin B12 was associated with decreased odds of depression compared to controls with scores > 9 (disordered), and intake of zinc increased its odds. In addition, consuming MUFA, vitamin B6, and black tea was associated with decreased odds of depression when comparing the reference group with cases under the threshold score of depression (normal); and consuming salt and zinc was associated with increased odds. Additionally, consuming vitamins A, B6, beta-carotene, and black tea was associated with decreased odds of depression when comparing the reference group with cases with scores > 9 (disordered), and consuming sugar and salt was associated with increased odds (Table 4).

Similar to the results for depression, considering controls under the threshold score of anxiety (normal) as the reference group, findings showed that consuming vitamin B3 was associated with decreased odds of anxiety compared to controls with scores > 7 (disordered). In addition, consuming vitamins B6, B12, and black tea was associated with decreased odds of anxiety when comparing the reference group with cases under the threshold score of anxiety (normal); and consuming salt and fat was associated with increased odds. In addition, consuming vitamins A, B6, and black tea was associated with decreased odds of anxiety when comparing the reference group with cases with scores > 7 (disordered), and consuming fat and salt was associated with increased odds (Table 4).

Moreover, considering controls under the threshold score of stress (normal) as the reference group showed that consuming sugar and coffee was associated with increased odds of stress compared to controls with scores > 14 (disordered). In addition, consuming beta-carotene, vitamins D, B6, and magnesium, and black tea was associated with decreased odds of stress when comparing the reference group with cases under the threshold score of the stress (normal); and consuming salt and SFA was associated with increased odds. Also, consuming vitamins A, D, and B6 was associated with decreased odds of stress when comparing the reference group with cases with scores > 14 (disordered), and consuming SFA, sugar, and salt was associated with increased odds (Table 4).

The highest significant negative association was between intake of salt and anxiety in cases under the threshold score of anxiety (OR = 4.899, 95% CI: 2.218–10.819; *p*-value < 0.001), and the highest significant protective effect was between intake of vitamin B6 and depression in cases with scores > 9 for depression (disordered) (OR = 0.132, 95% CI: 0.055–0.320; *p*-value < 0.001).

#### 3.5.2. Logistic Regression Models

Results of logistic regression models without any adjustment (crude model, A); adjusted for age and gender (B); and full model (C) adjusted for age, gender, total energy intake, BMI, regular physical activity, smoking, cancer history in immediate family members, marital and education status, *H. pylori* infection, aspirin or NSAID consumption, alcohol consumption between gastric cancer (outcome, binary variable) and depression, anxiety, and stress scores as continuous variables are represented in Table 5. According to logistic regression in all three models, there was a significant association between depression and increased odds of gastric cancer. However, there was no significant association (in any of the models) between anxiety and the odds of gastric cancer; in the fully adjusted model (C), there was a significant association between stress and increased odds of gastric cancer.

## 4. Discussion

Our study highlighted a significant association between depression and stress and increased gastric cancer odds. Having depression and stress significantly increased gastric cancer odds by 1.9 and 2.6 times (in a fully adjusted model), respectively. In addition, a significant association was observed between the intake of several nutrients and stress, anxiety, and depression in gastric cancer versus the control group. In fully adjusted multinomial logistic regressions, a one milligram (equivalent to 77% of RDA) increment of vitamin B6 significantly reduced the odds of having depression by 87%, anxiety by 78%, and stress by 77% in the gastric cancer subgroups. On the opposite side, 100 gram intake of sugar significantly increased the odds of depression by 1.9 times, and one gram of salt significantly increased the odds of depression by 2.5 times, anxiety by 3.7 times, and stress by 4.1 times in gastric cancer subgroups. In addition, significant protective results against stress were observed for MUFA, black tea, vitamins A, D, E, B12, and beta-carotene, and adverse effects for proteins, SFA, and caffeine intake in different subgroups.

Although the prevalence of psychological stress, depression, and anxiety in patients with gastric cancer has been reported to be high in earlier studies [17,18], the molecular mechanisms underlying depression-induced gastric cancer progression remain poorly understood, and limited studies could demonstrate a causal relationship between them, also considering nutritional factors. A recent study showed that depression might accelerate gastric cancer development through reactive oxygen species-activated tyrosine-protein kinase (ABL1) [25]. It was hypothesized that oxidative stress acts as a primary crosslink between gastric cancer and depression and that depressive patients show decreased levels of antioxidants, including catalase and superoxide dismutase (SOD), as well as glutathione peroxidase (GPX) [25]. ABL1 might be directly regulated by ROS-related methylation or *micro RNA 203* (*miR-203*), or the ROS-activated ligands of ABL1 may promote the activation of ABL1 indirectly [25]. Our results, showing higher values for TAC and MDA in patients with gastric cancer versus controls, support the hypothesis that oxidative stress may play a role in the causal pathway linking gastric cancer and mental health.

It has also been shown that stress hormone-induced activation of the β-adrenergic receptor2 (ADRB) signaling pathway plays a crucial role in gastric cancer metastasis/progression [26]. Moreover, ADRB2 antagonists suppressed proliferation/invasion/metastasis by inhibiting the ERK1/2-JNK-MAPK pathway and transcription factors, such as NF-κB, STAT3, AP-1, and CREB [26]. The results of our study concerning the significantly high level of inflammatory cytokines such as TNF-α and IL-6 and low levels of IL-10 in cases compared to controls highlights the importance of their role in the relation between gastric cancer and mental health, and such measures would be important for further mechanistic insights.

Stress has also been shown to suppress some facets of immune function, such as antigen presentation, T-cell proliferation, and humoral and cell-mediated immunity, mainly by releasing catecholamine and/or glucocorticoid hormones, and is related to higher inflammatory indicators [27]. A meta-analysis of 165 studies, including incidence/survival/mortality cohorts, indicated that stress-related psychosocial factors were associated with a higher cancer incidence, including gastric cancer [28]. Stress-prone personalities, unfavorable coping styles, negative emotional responses, or poor quality of life were related to higher cancer incidence, poorer cancer survival, and higher cancer mortality, suggesting adverse effects of stress-related psychosocial factors on cancer incidence and survival [28]. In this regard, an important topic regarding the association between mental health and gastric cancer is the relationship between psychoneuroimmunology and cancer, though studies in this area are very limited. A study reviewed that a likely mechanism linking psychosocial outcomes to cancer incidence/progression is dysregulated immune function; e.g., stress can suppress cellular immune function and enhance inflammation [29]. In addition, the catecholamines norepinephrine and epinephrine can promote tumor cell proliferation, pro-inflammatory cytokine secretion such as IL-6 and IL-8, and modulate vascular endothelial growth factor (VGEF), which could downregulate the expression of DNA repair genes in addition to altering natural killer (NK) cell activity [29]. This evidence is in line with the results of our study, showing a significant difference between inflammatory and oxidative stress biomarkers such as hs-CRP, IL-6, TNF-α, and MDA between cases and controls, as well as within mental health subgroups. Chronic inflammation measured based on TNF-a, CRP, and IL-6, among others, is highly correlated with an increased risk of gastric cancer; those measurements can be practical in predicting gastric cancer development at very early stages [30].

From a nutritional point of view, our study showed a significant association between vitamin B6 intake and the odds of increased depression, anxiety, and stress in gastric cancer and control subgroups. There is strong evidence that vitamin B6 plays an important role in preventing/controlling depression, anxiety, and stress symptoms [31,32]. From a mechanistic point of view, vitamin B6 is a cofactor that can affect depression by affecting neurotransmitters such as serotonin and norepinephrine in the brain, as vitamin B6 is required for synthesizing serotonin from tryptophan and the synthesis of norepinephrine from tyrosine [32]. In addition, vitamin B6 plays an active role in plasma homocysteine concentration, decarboxylation reactions, and transamination reactions [32]. A study has shown that individuals with anxiety and depression had a lower intake of vitamin B6 than healthy individuals [33]. Another study in adults reported an inverse relationship between vitamin B6 intake and perceived stress [34].

On the other hand, the association between low intake of vitamin B6 and the odds/risk of gastric cancer is well recognized [24,35]. However, additional prospective studies are needed to clarify the effect of vitamin B6 in the one-carbon metabolism pathway on the risk of developing gastric cancer. Extensive studies that assess the interrelation between vitamin B6 intake, genetic polymorphism in the vitamin B6 pathway, and other factors such as alcohol and smoking would be particularly valuable. More studies are needed regarding the role of vitamin B6 supplementation or intake via natural rich sources, including dark leafy greens, bananas, papayas, oranges, and cantaloupe, and the odds/risk of gastric cancer or depression, anxiety, and stress.

Similar to our results, the association between MUFA, vitamins A, E, beta-carotene, and black tea and reduced risk of depression, anxiety, and stress have been investigated in several studies [36,37,38,39,40,41], and the intake of these nutrients has been generally shown to reduce the risk of gastric cancer [42,43,44,45]. As demonstrated in this study, the levels of oxidative stress and inflammatory biomarkers in patients with gastric cancer were significantly higher than in controls and mental health subgroups. The effects of these nutrients may rely on their direct antioxidant effects and their anti-cancer activity by reducing NADPH oxidase-mediated production of ROS, NF-κB activation, and NF-κB-regulated TRAF1/2 gene expression and anti-inflammatory effects at the molecular/cellular level [46,47,48].

Although the role of vitamin D intake in reducing the risk or management of depression and anxiety has been shown [49,50,51], studies examining its association with physiological stress are scant. Peculiarly, we were only able to demonstrate its association with stress. Given the promising results of our study, despite the relatively small sample size, this issue deserves further investigation.

Contrarily, the results of our study showed that intake of salt, sugar, fat, SFA, and, unexpectedly, zinc significantly increased the odds of gastric cancer in subgroups with depression, anxiety, and stress. However, the high correlation between red meat and zinc intake can probably explain these unexpected results. Regarding salt and fats, in a systematic review, we showed that their intake was among the major risk factors for gastric cancer [5]. However, the precise mechanisms by which salt increases gastric cancer have remained unclear; the association is independent of *H. pylori* infection, smoking, tumor location, and histological type of gastric cancer [52]. Studies have also demonstrated a link between high salt intake and depression, anxiety, and stress [53,54,55]. The opposite pathway, such as having stress and increasing salt intake, has also been considered and reported [56]. It has been hypothesized that cortisol might be a critical factor in choosing high-salt foods. One possible explanation is that sympathoadrenal medullary system activity may increase urinary sodium excretion, resulting in a sodium-depleted state that may increase the person’s appetite for sodium [56]. Therefore, there is a need for future studies investigating the influence of chronic stress on eating behaviors, including the intake of salt/salty foods or other unhealthy food items such as fast foods and sugary products/sugar.

Some studies have highlighted an association between sugar intake and the risk of gastric cancer [57]. One prospective cohort study investigated sugar-sweetened beverage consumption and the subsequent risk of gastric cancer, though they did not observe a significant effect [58]. However, studies have shown that insulin resistance, a low HOMA-IR, and hyperglycemia were associated with gastric cancer [59,60]. A meta-analysis of observational studies has highlighted that the intake of sugar-sweetened beverages was significantly associated with a risk of depression, and a nonlinear dose–response relationship was found [61]. In addition, another meta-analysis of prospective studies concluded that higher intake of sugar-sweetened beverages significantly increased the risk of depression, cancer, and all-cause mortality compared to none or lower sugar-sweetened beverage consumption and that effects depended on participant characteristics such as age, BMI, and total energy intake [62]. Overconsumption of added sugars in particular can contribute to obesity and inflammation, which are important risk factors for gastric cancer and mental health, including depression [63,64]. In addition, a meta-analysis of observational studies highlighted that a high intake of sugar-sweetened beverages is significantly associated with a high risk of depression [61]. How sugar can cause depression requires further investigation; however, several mechanisms, such as imbalanced levels of insulin and blood glucose (insulin resistance), effects on thyroid hormones, deviation/dysregulation in the mesolimbic dopamine system related to short-term boosts in mood through longer-term adverse effects, increased levels of inflammatory and oxidative stress biomarkers, alterations in the gut–brain axis, and increased production of toxic molecules such as advanced glycation end-products (AGEs) have been proposed [65]. Though the results investigating the association between sugar intake and depression are rather convincing, very limited studies have investigated the potential relationship between their intake and stress and anxiety.

Therefore, according to the results of our study, as well as the findings of previous studies, recommending adherence to healthy dietary patterns, such as the Mediterranean diet, and dietary recommendations, such as the Healthy Eating Index or the Dietary Approaches to Stop Hypertension, and as a result, receiving high amounts of fruits, vegetables, and whole grains rich in fiber, minerals and vitamins such as A, B6, and fish and sea products rich in PUFA and vitamin D and limiting the intake of processed meats with high salt and SFA and trans fatty acid content and beverages with high sugar in addition to helping improving mental health will also help to reduce the odds of developing gastric cancer.

One of the most important strengths of our study was the use of incidence cases; thus, the study design allowed us to examine the chronology of factors/risks to some extent. For example, a valid FFQ examined the participants’ eating habits in the previous year (the year before official diagnosis), and food intake was examined independently of the odds of mental disorders and gastric cancer. The same was the case for DASS, so the participants were asked to specify their signs and symptoms before the diagnosis so that we could minimize the possibility of the diagnosis effect in the final score. However, as a general limitation of retrospective studies, recall bias for FFQ and DASS can be generalized to our study. To minimize this limitation, we used a trained expert to collect the data, and the fact that the participants were incidence cases was also helpful. Another limitation of our study, due to its case–control design, is that with this design, we could clearly determine the causal relationships and only predicted the odds of the event, thus prospective and interventional studies are required.

Our study findings strives to engage a diverse audience, including medical professionals, researchers, public health practitioners, patients, caregivers, and educational institutions. Our research outcomes will have broader scientific implications, including the potential for tailored prevention strategies, holistic patient care, and targeted public health initiatives. This work will pave the way for subsequent research, including longitudinal studies, mechanistic investigations, intervention studies, and exploration of genetic and environmental factors, all of which will contribute to a more nuanced understanding of the intricate association between mental health and dietary factors in gastric cancer odds. This, in turn, will inform more effective prevention and treatment strategies.

## 5. Conclusions

Our results showed that depression and stress significantly increased the odds of developing gastric cancer. In addition, dietary intake of vitamins B6, D, and A, beta-carotene, MUFA, and black tea decreased, and the consumption of total fat, sugar, and salt increased the odds of gastric cancer in subgroups of depression, stress, and also anxiety, respectively. Considering that cases consumed significantly lower amounts of nutrients, including vitamins B6, A, and D, but more total fat, SFA, salt, and sugar compared to controls, advising that people follow healthy dietary patterns would be expected to have beneficial effects with respect to gastric cancer risk. Conducting prospective studies to understand the chronology of associations better, specify causal relationships, and clarify underlying mechanisms seems imperative.

## Figures and Tables

**Table 1:** The severity (scores) of each subscale of the Depression, Anxiety, and Stress Scale *.

Severity	Stress		Anxiety		Depression	
Normal	0–14	Normal	0–7	Normal	0–9	Normal
Mild	15–18	Disordered	8–9	Disordered	10–13	Disordered
Average	19–25		10–14		14–20	
Severe	26–33		15–19		21–27	
Very severe	≥34		≥20		≥28	

* For each subscale, we considered the “Normal” categories as “Normal” and all other categories as “Disordered”.

**Table 2:** Comparison of baseline characteristics of controls and cases (number and frequencies in percentage).

Characteristics	Cases (*n* = 82)	Controls (*n* = 95)	Total (*n* = 177)	*p*-Value ^a,^**
**Depression**				0.170
Normal	43 (52.4%)	34 (35.7%)	77 (43.5%)	
Mild	17 (20.7%)	28 (29.4%)	45 (25.4%)	
Average	17 (20.7%)	25 (26.3%)	42 (23.7%)	
Severe	5 (6.1%)	8 (8.4%)	13 (7.3%)	
Very severe	0	0	0	
**Anxiety**				0.318
Normal	25 (30.4%)	38 (40.0%)	63 (35.5%)	
Mild	13 (15.8%)	7 (7.3%)	20 (11.2%)	
Average	28 (34.1%)	36 (37.9%)	64 (36.1%)	
Severe	11 (13.4%)	10 (10.5%)	21 (11.8%)	
Very severe	5 (6.1%)	4 (4.2%)	9 (5.1%)	
**Stress**				0.563
Normal	64 (78.0%)	81 (85.2%)	145 (81.9%)	
Mild	8 (9.7%)	5 (5.2%)	13 (7.3%)	
Average	5 (6.1%)	4 (4.2%)	9 (5.1%)	
Severe	4 (4.8%)	5 (5.2%)	9 (5.1%)	
Very severe	1 (1.2%)	0	1 (0.5%)	
**Gender**				0.985
Men	45 (54.8%)	52 (54.7%)	97 (54.8%)	
Women	37 (45.1%)	43 (45.2%)	80 (45.1%)	
**Residency**				0.283
Urban	60 (73.1%)	76 (80.0%)	136 (76.8%)	
Rural	22 (26.8%)	19 (20.0%)	41 (23.1%)	
**Smoking (yes)**	14 (17.1%)	15 (15.7%)	29 (16.3%)	0.818
**Alcohol (yes)**	11 (13.4%)	9 (9.4%)	20 (11.2%)	0.409
**Education**				0.241
Diploma and Low Literate	51 (62.1%)	67 (70.5%)	118 (66.6%)	
Higher Than Diploma	31 (37.8%)	28 (29.4%)	59 (33.3)	
**Marital status**				0.622
Single	16 (19.5%)	14 (14.7%)	30 (16.9%)	
Married	61 (74.3%)	73 (76.8%)	134 (75.7%)	
Separated/Divorced	5 (6.1%9	8 (8.4%)	13 (7.3%)	
**Regular physical activity (yes)**	14 (17.1%)	30 (31.5%)	44 (24.8%)	**0.026**
Cancer history (yes) *	13 (15.8%9	11 (11.5%)	24 (13.5%)	0.407
***H. pylori***				**0.002**
Positive	61 (74.3%)	49 (51.5%)	110 (62.1%)	
Negative	21 (25.6%)	46 (48.2%)	67 (37.8%)	
**Aspirin Or NSAIDs ^$^ intake (yes)**	7 (8.5%)	9 (9.4%)	16 (9.1%)	0.828

^a^ Chi-square (χ^2^) test was used to obtain *p*-values. * Cancer history in immediate family members. ** Benjamini–Hochberg correction was applied to all *p*-values: all *p*-values are displayed after this correction. Significant values are given in bold. ^$^ NSAID: non-steroidal anti-inflammatory drugs.

**Table 3:** Comparison (mean ±SD) of inflammatory and oxidative stress markers of controls and cases and depression, anxiety, and stress groups ^f,^*.

	Cases (*n* = 82)	Controls (*n* = 95)	*p*-Value	Depression		*p*-Value	Anxiety		*p*-Value	Stress		*p*-Value
				˃9 (*n* = 100)	≤9 (*n* = 77) (Normal)		˃7 (*n* = 114)	≤7 (*n* = 63) (Normal)		˃14 (*n* = 32)	≤14 (*n* = 145) (Normal)	
hs-CRP (mg/L)	2.39 ± 1.10	1.82 ± 1.01	**0.001**	2.23 ± 1.22	1.97 ± 0.96	0.118	2.13 ± 1.15	2.00 ± 0.96	0.433	2.05 ± 0.85	2.09 ± 1.14	0.865
IL-6 (pg/mL)	234.8 ± 172.6	102.8 ± 61.3	**<0.001**	189.1 ± 184.6	144.5 ± 93.1	**0.038**	167.4 ± 153.9	157.8 ± 117.3	0.666	176.8 ± 108.6	161.1 ± 148.2	0.573
IL-1β (pg/mL)	2.84 ± 1.52	1.75 ± 0.88	**<0.001**	2.31 ± 1.35	2.21 ± 1.32	0.643	2.35 ± 1.36	2.07 ± 1.27	0.189	2.51 ± 1.42	2.21 ± 1.31	0.233
TNF-α (pg/mL)	42.0 ± 29.1	19.1 ± 16.1	**<0.001**	33.3 ± 32.8	26.9 ± 18.1	0.102	29.8 ± 25.5	29.4 ± 26.2	0.925	39.4 ± 32.5	27.6 ± 23.5	**0.018**
IL-10 (pg/mL)	1.94 ± 1.40	2.96 ± 2.00	**<0.001**	2.11 ± 1.44	2.79 ± 2.01	**0.012**	2.44 ± 1.87	2.57 ± 1.71	0.640	2.82 ± 1.76	2.41 ± 1.82	0.251
IL-4 (pg/mL)	11.46 ± 12.4	9.01 ± 7.18	0.119	9.51 ± 9.63	10.64 ± 10.33	0.454	10.01 ± 8.79	10.38 ± 12.00	0.813	7.98 ± 6.89	10.62 ± 10.54	0.177
TAC (mmol/L)	1.16 ± 1.26	1.91 ± 1.27	**<0.001**	1.61 ± 1.53	1.53 ± 1.14	0.730	1.56 ± 1.51	1.57 ± 1.21	0.983	1.92 ± 1.94	1.48 ± 1.14	0.093
MDA (μmol/L)	3.72 ± 2.19	3.01 ± 1.68	**0.017**	3.30 ± 1.96	3.36 ± 1.97	0.842	3.56 ± 2.11	2.94 ± 1.59	**0.044**	3.54 ± 1.93	3.29 ± 1.97	0.521
FBG (mg/dL)	97.5 ± 36.2	89.4 ± 13.2	**0.046**	98.3 ± 36.7	89.3 ± 14.2	**0.026**	94.2 ± 28.0	91.2 ± 24.2	0.479	96.5 ± 33.5	92.4 ± 25.1	0.435

^f^ Independent samples *t*-test is used to compare two groups’ means. * Benjamini–Hochberg correction was applied to all *p*-values: all *p*-values are displayed after this correction. Significant values are given in bold. hsCRP = high-sensitivity c-reactive protein, IL = interleukin, TNF-α = tumor necrosis factor alpha, TAC = total antioxidant capacity, MDA = malondialdehyde, FBG = fasting blood glucose.

**Table 4:** Fully adjusted multinomial logistic regression (ORs (95%CI); *p*-values for trends) between dietary intakes (macro- and micronutrients) and subgroups of depression, anxiety, and stress (normal vs. elevated) * ^ꝉ^.

Dietary Intakes	Depression				Anxiety				Stress			
	Controls ≤9 Score (*n* = 34) ^a^	Controls ˃9 Score (*n* = 61) ^b^	Cases ≤9 Score (*n* = 43) ^c^	Cases ˃9 Score (*n* = 39) ^d^	Controls ≤7 Score (*n* = 38) ^e^	Controls ˃7 Score (*n* = 57) ^f^	Cases ≤7 Score (*n* = 25) ^g^	Cases ˃7 Score (*n* = 57) ^k^	Controls ≤14 Score (*n* = 81) ^l^	Controls ˃14 Score (*n* = 14) ^m^	Cases ≤14 Score (*n* = 64) ^n^	Cases ˃14 Score (*n* = 18) ^o^
Protein (g/d)	Ref.	1.002 (0.991–1.012); 0.618	**4.149 (1.392–12.364); 0.001**	0.998 (0.986–1.010); 0.747	Ref.	0.994 (0.984–1.004); 0.251	0.997 (0.984–1.010); 0.672	0.991 (0.980–1.003); 0.132	Ref.	0.985 (0.967–1.003); 0.111	0.997 (0.987–1.006); 0.456	0.991 (0.972–1.009); 0.324
Carbohydrate (g/d)	Ref.	1.002 (0.998–1.006); 0.375	1.003 (0.998–1.007); 0.248	1.003 (0.998–1.007); 0.214	Ref.	0.997 (0.993–1.000); 0.082	1.000 (0.996–1.004); 0.994	0.999 (0.996–1.003); 0.697	Ref.	1.002 (0.997–1.007); 0.518	1.003 (0.999–1.006); 0.131	1.002 (0.997–1.008); 0.430
Fat (g/d)	Ref.	0.989 (0.976–1.003); 0.119	1.003 (0.991–1.016); 0.586	1.004 (0.991–1.017); 0.511	Ref.	1.009 (0.995–1.023); 0.222	**1.017 (1.002–1.033); 0.029**	**1.015 (1.001–1.030); 0.033**	Ref.	0.987 (0.965–1.009); 0.255	1.007 (0.997–1.017); 0.196	1.010 (0.996–1.024); 0.181
SFA (g/d)	Ref.	1.001 (0.983–1.019); 0.916	1.015 (0.997–1.033); 0.096	1.013 (0.995–1.032); 0.150	Ref.	1.003 (0.986–1.022); 0.704	1.017 (0.999–1.036); 0.068	1.016 (0.999–1.034); 0.063	Ref.	1.006 (0.984–1.029); 0.570	**1.014 (1.001–1.027); 0.050**	**1.018 (1.002–1.035); 0.041**
MUFA (g/d)	Ref.	**0.949 (0.909–0.990); 0.016**	**0.955 (0.911–0.999); 0.049**	0.967 (0.925–1.010); 0.127	Ref.	0.994 (0.955–1.035); 0.777	0.986 (0.937–1.037); 0.573	0.991 (0.951–1.032); 0.656	Ref.	0.971 (0.915–1.031); 0.341	0.992 (0.958–1.026); 0.630	0.940 (0.879–1.006); 0.072
PUFA (g/d)	Ref.	0.984 (0.964–1.005); 0.139	0.976 (0.951–1.001); 0.061	0.987 (0.964–1.010); 0.255	Ref.	1.020 (0.996–1.045); 0.107	1.013 (0.985–1.043); 0.370	0.999 (0.974–1.026); 0.976	Ref.	0.958 (0.914–1.004); 0.074	0.984 (0.965–1.003); 0.091	0.991 (0.956–1.027); 0.607
Vit A (µg/d)	Ref.	0.999 (0.998–1.000); 0.134	0.999 (0.997–1.000); 0.158	**0.997 (0.996–0.999); 0.006**	Ref.	0.999 (0.998–1.000); 0.111	0.999 (0.997–1.001); 0.182	**0.998 (0.997–0.999); 0.012**	Ref.	0.999 (0.998–1.001); 0.640	0.999 (0.998–1.000); 0.193	**0.997 (0.994–0.999); 0.044**
Beta–carotene (µg/d)	Ref.	0.999 (0.999–1.000); 0.333	0.999 (0.999–1.000); 0.253	**0.999 (0.998–0.999); 0.043**	Ref.	0.999 (0.999–1.000); 0.213	0.999 (0.999–1.000); 0.117	0.999 (0.999–1.000); 0.064	Ref.	0.999 (0.998–0.999); 0.222	**0.999 (0.999–0.999); 0.004**	0.999 (0.999–1.000); 0.219
Vit D (µg/d)	Ref.	1.133 (0.875–1.467); 0.342	0.878 (0.645–1.196); 0.409	0.839 (0.611–1.154); 0.281	Ref.	1.104 (0.861–1.415); 0.436	0.912 (0.654–1.271); 0.856	0.804 (0.603–1.073); 0.139	Ref.	0.913 (0.630–1.324); 0.631	**0.877 (0.697–0.958); 0.050**	**0.443 (0.237–0.828); 0.011**
Vit E (mg/d)	Ref.	0.964 (0.908–1.023); 0.222	0.999 (0.942–1.062)	1.012 (0.955–1.072); 0.692	Ref.	1.010 (0.951–1.073); 0.740	1.027 (0.957–1.102); 0.457	1.037 (0.976–1.102); 0.239	Ref.	0.949 (0.867–1.038); 0.248	0.990 (0.940–1.043); 0.708	1.074 (0.997–1.157); 0.061
Vit C (mg/d)	Ref.	1.001 (0.994–1.007); 0.864	0.996 (0.989–1.004); 0.325	1.001 (0.994–1.008); 0.842	Ref.	1.003 (0.996–1.009); 0.429	0.996 (0.988–1.005); 0.397	1.002 (0.995–1.008); 0.650	Ref.	0.997 (0.988–1.007); 0.574	0.997 (0.992–1.003); 0.351	0.998 (0.990–1.006); 0.633
Thiamin (mg/d)	Ref.	1.510 (0.910–2.507); 0.111	0.977 (0.547–1.745); 0.937	0.917 (0.508–1.654); 0.777	Ref.	0.862 (0.555–1.340); 0.511	0.807 (0.463–1.405); 0.444	0.637 (0.393–1.031); 0.066	Ref.	1.109 (0.589–2.086); 0.750	0.788 (0.518–1.199); 0.266	0.594 (0.253–1.390); 0.594
Riboflavin (mg/d)	Ref.	0.743 (0.431–1.281); 0.285	0.560 (0.292–1.073); 0.081	0.802 (0.444–1.438); 0.459	Ref.	0.983 (0.576–1.674–0.948	1.004 (0.515–1.957); 0.992	0.702 (0.390–1.261); 0.236	Ref.	1.703 (0.848–3.418); 0.134	0.952 (0.586–1.546); 0.842	0.576 (0.237–1.401); 0222
Niacin (mg/d)	Ref.	0.985 (0.945–1.026); 0.467	0.986 (0.942–1.032); 0.536	1.017 (0.974–1.063); 0.444	Ref.	**0.952 (0.914–0.994); 0.024**	0.987 (0.940–1.036); 0.587	0.981 (0.942–1.022); 0.360	Ref.	1.014 (0.958–1.072); 0.632	1.011 (0.976–1.047); 0.555	1.001 (0.944–1.061); 0.983
Vit B6 (mg/d)	Ref.	0.888 (0.592–1.335); 0.570	**0.396 (0.205–0.764); 0.006**	**0.132 (0.055–0.320); <0.001**	Ref.	0.821 (0.555–1.212); 0.321	**0.249 (0.109–0.566); 0.001**	**0.226 (0.117–0.435); <0.001**	Ref.	1.166 (0.674–2.017); 0.582	**0.306 (0.168–0.558); <0.001**	**0.216 (0.070–0.664); 0.007**
Folate (µg/d)	Ref.	0.999 (0.997–1.001); 0.427	0.999 (0.997–1.001); 0.287	0.999 (0.996–1.001); 0.167	Ref.	0.999 (0.998–1.002); 0.831	0.999 (0.997–1.001); 0.417	0.999 (0.997–1.001); 0.362	Ref.	1.002 (0.999–1.004); 0.074	0.999 (0.998–1.001); 0.689	0.999 (0.997–1.002); 0.742
Vit B12 (µg/d)	Ref.	**0.885 (0.734–0.996); 0.044**	0.903 (0.782–1.042); 0.162	1.034 (0.904–1.183); 0.623	Ref.	0.925 (0.791–1.081); 0.326	**0.803 (0.644–0.999); 0.050**	1.073 (0.932–1.235); 0.333	Ref.	1.095 (0.906–1.232); 0.348	1.053 (0.922–1.202); 0.444	1.116 (0.950–1.310); 0.183
Magnesium (mg/d)	Ref.	0.999 (0.997–1.003); 0.956	0.999 (0.996–1.002); 0.561	0.998 (0.995–1.001); 0.189	Ref.	1.002 (0.999–1.005); 0.236	0.999 (0.996–1.003); 0.848	0.999 (0.997–1.003); 0.790	Ref.	0.997 (0.993–1.001); 0.205	**0.997 (0.995–0.999); 0.041**	1.001 (0.997–1.005); 0.731
Zinc (mg/d)	Ref.	**1.176 (1.062–1.302); 0.002**	**1.176 (1.054–1.313); 0.004**	1.076 (0.964–1.200); 0.191	Ref.	0.930 (0.858–1.009); 0.080	0.936 (0.843–1.038); 0.210	0.982 (0.909–1.062); 0.653	Ref.	0.980 (0.873–1.101); 0.736	0.996 (0.928–1.068); 0.902	1.006 (0.901–1.123); 0.911
Selenium (µg/d)	Ref.	1.003 (0.994–1.013); 0.5165	1.004 (0.994–1.015); 0.426	0.991 (0.979–1.003); 0.126	Ref.	0.998 (0.989–1.007); 0.699	0.994 (0.982–1.006); 0.310	0.997 (0.988–1.007); 0.563	Ref.	1.006 (0.994–1.018); 0.354	0.995 (0.987–1.004); 0.253	1.007 (0.995–1.019); 0.250
Sugar (g/d)	Ref.	1.009 (0.998–1.020); 0.113	1.011 (0.999–1.023); 0.077	**1.017 (1.005–1.029); 0.005**	Ref.	0.999 (0.988–1.009); 0.823	1.008 (0.996–1.020); 0.188	1.008 (0.997–1.018); 0.151	Ref.	**1.012 (1.001–1.025); 0.050**	1.007 (0.998–1.016); 0.156	**1.022 (1.009–1.036); <0.001**
Salt (g/d)	Ref.	0.732 (0.369–1.453); 0.372	**3.045 (1.481–6.262); 0.002**	**2.581 (1.263–5.273); 0.009**	Ref.	1.310 (0.665–2.582); 0.434	**4.899 (2.218–10.819); <0.001**	**3.759 (1.862–7.595); <0.001**	Ref.	2.359 (0.998–5.662); 0.059	**3.787 (2.036–7.044); <0.001**	**4.132 (1.872–9.121); <0.001**
Fiber (g/d)	Ref.	1.011 (0.987–1.036); 0.358	1.002 (0.975–1.030); 0.874	0.999 (0.972–1.026); 0.929)	Ref.	0.992 (0.970–1.016); 0.514	0.987 (0.958–1.017); 0.404	0.989 (0.966–1.013); 0.380	Ref.	0.993 (0.959–1.028); 0.682	0.994 (0.974–1.014); 0.555	0.981 (0.942–1.021); 0.347
Caffeine (mg/d)	Ref.	0.999 (0.995–1.004); 0.784	1.000 (0.995–1.005); 0.974	1.000 (0.996–1.005); 0.962	Ref.	0.998 (0.993–1.002); 0.286	1.002 (0.997–1.006); 0.491	0.997 (0.993–1.002); 0.233	Ref.	**1.006 (1.001–1.011); 0.020**	1.002 (0.999–1.006); 0.216	1.001 (0.994–1.008); 0.777
Black tea (mL/d)	Ref.	0.999 (0.999–1.000); 0.124	**0.999 (0.998–0.999); 0.009**	**0.999 (0.999–0.999); 0.030**	Ref.	0.999 (0.999–1.000); 0.140	**0.998 (0.996–0.999); 0.050**	**0.999 (0.998–0.999); 0.006**	Ref.	1.001 (0.999–1.001); 0.083	**0.999 (0.997–0.999); 0.050**	0.999 (0.998–1.001); 381

^a^ Cases with ≤9 score for depression (normal); ^b^ controls with ≤9 score for depression (normal); ^c^ cases with ˃9 score for depression (having depression); ^d^ controls with ˃9 score for depression (having depression); ^e^ cases with ≤7 score for anxiety (normal); ^f^ controls with ≤7 score for anxiety (normal); ^g^ cases with ˃7 score for anxiety (having anxiety); ^k^ controls with ˃7 score for anxiety (having anxiety); ^l^ cases with ≤14 score for the stress (normal); ^m^ controls with ≤14 score for the stress (normal); ^n^ cases with ˃14 score for stress (having stress); ^o^ controls with ˃14 score for the stress (having stress); ^ꝉ^ multinomial logistic regression models adjusted for age, gender, total energy intake, BMI, regular physical activity (yes/no), smoking (yes/no), cancer history in immediate family members (yes/no), marital and education status, *H. pylori* infection, aspirin or NSAID consumption (yes/no), alcohol consumption (yes/no) were used to report ORs and 95% CI; * significant values are given in bold. Ref = reference (controls under the threshold score of the disorder (depression, anxiety, stress) were considered the reference group). SFA = saturated fatty acids; MUFA = monounsaturated fatty acids; PUFA = polyunsaturated fatty acids; Vit = vitamin; µg = microgram; OR = odds ratio; CI = confidence interval, BMI = body mass index.

**Table 5:** Logistic regression (ORs (95%CI)) between gastric cancer and depression, anxiety, and stress *.

Models	Depression			Anxiety			Stress		
	Controls (*n* = 95)	Cases (*n* = 82)	*p*-Value	Controls (*n* = 95)	Cases (*n* = 82)	*p*-Value	Controls (*n* = 95)	Cases (*n* = 82)	*p*-Value
Model A	Ref.	**1.978 (1.082–3.616)**	**0.027**	Ref.	1.520 (0.814–2.837)	0.189	Ref.	1.627 (0.752–3.520)	0.216
Model B	Ref.	**2.022 (1.098–3.724)**	**0.024**	Ref.	1.473 (0.782–2.776)	0.231	Ref.	1.654 (0.756–3.618)	0.207
Model C	Ref.	**1.938 (1.009–3.723)**	**0.047**	Ref.	1.054 (0.731–3.095)	0.267	Ref.	**2.630 (1.014–6.819)**	**0.047**

A: Logistic regression without any adjustment (crude model). B: Logistic regression models adjusted for age and gender. C: Logistic regression models adjusted for age, gender, total energy intake, BMI, regular physical activity (yes/no), smoking (yes/no), cancer history in immediate family members (yes/no), marital and education status, *H. pylori* infection, aspirin or NSAID consumption (yes/no), alcohol consumption (yes/no) were used to report ORs and 95% CI. Ref = Reference (controls were considered as the reference group). * Significant values are given in bold.

## Data Availability

The original contributions presented in this study are included in this article and Supplementary Materials. Further inquiries can be directed to the corresponding author.

## Supplementary Materials

The following supporting information can be downloaded at: https://www.mdpi.com/article/10.3390/nu15234981/s1; Table S1: Comparison (mean ± SD) of dietary intakes (macro- and micronutrients) of participants in depression subgroups—here with normal vs. elevated degrees of depression; Table S2: Comparison (mean ± SD) of dietary intakes (macro- and micronutrients) of participants in anxiety subgroups—here with normal vs. elevated levels of anxiety; Table S3: Comparison (mean ± SD) of dietary intakes (macro- and micronutrients) of participants in stress subgroups—here with normal vs. elevated levels of stress; Table S4: Pearson correlation coefficients and *p*-values between dietary intakes (macro- and micronutrients) and scores of depression, anxiety, and stress in cases, controls, and total sample.
