# Supplementary material for: Mental Health Conditions, Including Depression and Stress, Are Associated with Increased Odds of Gastric Cancer—Insights into the Role of Diet: A Case-Control Study
Source: Nutrients. 2023 Nov 30;15(23):4981. doi: 10.3390/nu15234981 (PMC10708069; doi:10.3390/nu15234981)
Supplement: Supplementary file 1 [file nutrients-15-04981-s001.zip › nutrients-2675503-supplementary.pdf]

nutrients

MDPI

Supplementary Material

Supplementary Table S1. Comparison (mean ± SD) of dietary intakes (macro- and micronutrients) of participants in depression subgroups – here with normal vs. elevated degrees of depression[f*].

**Dietary intakes**	**Cases ≤9 score (n=43)**[a]	**Controls ≤9 score (n=34)**[b]	***p*-value** (a *vs.* b)	**Cases >9 score (n=39)**[c]	**Controls >9 score (n=61)**[d]	***p*-value** (c *vs.* d)	***p*-value** (a *vs.* c)	***p*-value** (b *vs.* d)	***p*-value** (b *vs.* c)	***p*-value** (a *vs.* d)	***p*-value** (Between Groups)
Energy intake (kcal/d)	3009 ± 639	2979 ± 507	0.996	3016 ± 618	2997 ± 574	0.999	0.999	0.999	0.993	0.999	0.994
Protein (g/d)	99.5 ± 42.8	107.1 ± 36.2	0.844	103.0 ± 35.2	110.7 ± 41.9	0.777	0.797	0.972	0.973	0.491	0.527
Carbohydrates (g/d)	375.8 ± 110.3	358.3 ± 93.1	0.914	385.5 ± 119.4	382.0 ± 130.0	0.999	0.982	0.779	0.754	0.993	0.755
Fat (g/d)	122.1 ± 46.9	113.0 ± 29.6	0.721	117.0 ± 38.3	103.0 ± 33.8	0.272	0.926	0.596	0.971	**0.056**	0.068
SFA (g/d)	57.1 ± 43.0	39.4 ± 23.8	0.088	48.7 ± 32.1	40.5 ± 28.3	0.605	0.653	0.999	0.614	0.055	**0.042**
MUFA (g/d)	29.0 ± 10.8	33.6 ± 10.6	0.221	29.7 ± 12.1	28.3 ± 8.6	0.900	0.988	0.082	0.394	0.985	0.111
PUFA (g/d)	26.1 ± 15.0	35.5 ± 22.5	0.131	30.2 ± 18.1	29.0 ± 19.2	0.990	0.754	0.374	0.625	0.860	0.180
Vit A (µg/d)	624.1 ± 193.6	756.3 ± 456.1	0.224	542.9 ± 207.6	663.1 ± 324.3	0.231	0.633	0.492	**0.019**	0.920	**0.030**
Beta-carotene (µg/d)	4750 ± 1806	5457 ± 2353	0.460	4416 ± 1834	5931 ± 2301	**0.003**	0.890	0.719	0.154	**0.027**	**0.002**
Vit D (µg/d)	1.9 ± 1.6	2.2 ± 1.2	0.893	1.8 ± 1.4	2.5 ± 1.7	0.130	0.989	0.743	0.755	0.230	0.114
Vit E (mg/d)	18.6 ± 8.1	19.7 ± 6.3	0.930	19.9 ± 10.1	18.0 ± 7.0	0.661	0.883	0.765	0.999	0.983	0.624
Vit C (mg/d)	147.0 ± 73.8	158.9 ± 48.0	0.861	162.6 ± 78.4	161.1 ± 60.1	0.999	0.714	0.999	0.996	0.708	0.682
Thiamin (mg/d)	2.1 ± 0.5	2.0 ± 0.7	0.932	2.0 ± 0.9	2.3 ± 1.0	0.149	0.930	0.278	0.996	0.601	0.131
Riboflavin (mg/d)	2.0 ± 0.6	2.3 ± 0.8	0.286	2.2 ± 0.6	2.1 ± 0.8	0.978	0.714	0.627	0.880	0.871	0.346
Niacin (mg/d)	28.1 ± 8.7	30.1 ± 13.4	0.829	31.7 ± 11.2	28.3 ± 8.9	0.375	0.378	0.847	0.911	0.999	0.342
Vit B6 (mg/d)	2.1 ± 0.5	2.8 ± 1.2	**0.003**	1.9 ± 0.4	2.7 ± 0.9	**<0.001**	0.505	0.929	**<0.001**	**0.004**	**<0.001**
Folate (µg/d)	673.3 ± 277.2	730.3 ± 205.7	0.722	652.7 ± 237.1	698.6 ± 222.7	0.782	0.979	0.924	0.505	0.951	0.528
Vit B12 (µg/d)	5.3 ± 3.2	6.0 ± 2.5	0.801	6.2 ± 4.6	4.8 ± 2.4	0.155	0.570	0.349	0.988	0.882	0.144
Magnesium (mg/d)	518.1 ± 154.3	542.2 ± 146.1	0.901	496.4 ± 157.9	541.4 ± 150.0	0.473	0.916	0.999	0.574	0.868	0.458
Zinc (mg/d)	12.7 ± 5.2	16.1 ± 4.6	**0.020**	14.0 ± 3.9	16.4 ± 5.7	0.110	0.241	0.635	**0.005**	**0.001**	**0.003**
Selenium (µg/d)	132.4 ± 52.6	124.5 ± 40.6	0.869	109.7 ± 40.3	130.9 ± 43.0	0.098	0.103	0.908	0.492	0.998	0.082
Sugar (g/d)	131.1 ± 45.8	117.8 ± 31.7	0.555	147.2 ± 54.9	133.1 ± 40.3	0.391	0.346	0.375	**0.025**	0.996	**0.045**
Salt (g/d)	2.6 ± 0.9	1.9 ± 0.4	**0.001**	2.3 ± 0.8	1.8 ± 0.6	**0.002**	0.540	0.912	**0.057**	**<0.001**	**<0.001**
Fiber (g/d)	44.8 ± 18.0	44.4 ± 16.6	0.999	44.0 ± 14.2	47.9 ± 19.3	0.689	0.996	0.785	0.999	0.808	0.647
Caffeine (mg/d)	132.1 ± 120.1	136.6 ± 92.4	0.997	138.5 ± 74.5	128.8 ± 96.7	0.962	0.991	0.982	0.999	0.998	0.963
Black tea (mL/d)	441.5 ± 360.8	991.4 ± 893.7	**0.003**	555.9 ± 616.8	646.0 ± 471.8	0.887	0.815	0.153	**0.050**	0.302	**0.006**

[a] Cases with ≤9 score for depression (normal); [b] Controls with ≤9 scores for depression (normal); [c] Cases with >9 score for depression (having depression)
[d] Controls with >9 score for depression (having depression); [f] One-way ANOVA with Tukey post hoc was used for the comparison. *Significant values are given in **bold**. SFA= saturated fatty acids; MUFA= monounsaturated fatty acids; PUFA= polyunsaturated fatty acids; Vit= vitamin.

nutrients

Supplementary Material

MDPI

Supplementary Table S2. Comparison (mean ± SD) of dietary intakes (macro- and micronutrients) of participants in anxiety subgroups – here with normal vs. elevated levels of anxiety [f]*.

**Dietary intakes**	**Cases ≤7 score (n=25)**[a]	**Controls ≤7 score (n=38)**[b]	***p*-value** (a *vs.* b)	**Cases >7 score (n=57)**[c]	**Controls >7 score (n=57)**[d]	***p*-value** (c *vs.* d)	***p*-value** (a *vs.* c)	***p*-value** (b *vs.* d)	***p*-value** (b *vs.* c)	***p*-value** (a *vs.* d)	***p*-value** (Between Groups)
Energy intake (kcal/d)	3091 ± 708.9	3078 ± 444.5	0.999	2978 ± 588.6	2933 ± 605.6	0.977	0.851	0.640	0.848	0.672	0.552
Protein (g/d)	106.4 ± 43.8	115.6 ± 40.5	0.803	98.8 ± 37.2	105.2 ± 39.1	0.824	0.855	0.954	0.183	0.999	0.253
Carbohydrates (g/d)	386.6 ± 118.5	399.5 ± 98.2	0.973	377.6 ± 113.0	356.1 ± 127.8	0.754	0.988	0.282	0.754	0.962	0.329
Fat (g/d)	123.0 ± 56.9	101.9 ± 25.8	0.137	118.2 ± 35.5	109.7 ± 36.2	0.625	0.954	0.757	0.169	0.462	0.093
SFA (g/d)	54.8 ± 49.9	39.1 ± 22.1	0.248	52.4 ± 32.2	40.8 ± 29.5	0.232	0.990	0.995	0.214	0.283	0.072
MUFA (g/d)	29.0 ± 11.3	30.5 ± 7.6	0.946	29.5 ± 11.4	30.0 ± 10.8	0.996	0.996	0.996	0.973	0.979	0.947
PUFA (g/d)	31.4 ± 15.1	27.3 ± 18.0	0.834	26.6 ± 17.1	34.0 ± 21.8	0.150	0.709	0.321	0.998	0.935	0.147
Vit A (μg/d)	616.8 ± 220.0	768.9 ± 419.7	0.222	571.7 ± 196.0	648.1 ± 341.7	0.548	0.928	0.242	**0.013**	0.974	**0.025**
Beta-carotene (μg/d)	4560 ± 1891	5417 ± 2088	0.392	4604 ± 1799	5991 ± 2451	**0.003**	0.999	0.651	0.256	**0.026**	**0.002**
Vit D (μg/d)	2.0 ± 1.8	2.2 ± 1.4	0.959	1.8 ± 1.4	2.5 ± 1.7	0.082	0.926	0.846	0.549	0.595	0.116
Vit E (mg/d)	18.9 ± 7.2	18.6 ± 7.9	0.999	19.3 ± 9.8	18.6 ± 6.4	0.966	0.995	0.999	0.966	0.999	0.960
Vit C (mg/d)	140.1 ± 44.6	154.3 ± 52.7	0.838	160.7 ± 85.8	164.4 ± 57.9	0.991	0.564	0.884	0.967	0.419	0.462
Thiamin (mg/d)	2.2 ± 0.7	2.3 ± 1.1	0.942	2.0 ± 0.7	2.2 ± 0.8	0.706	0.854	0.916	0.376	0.999	0.430
Riboflavin (mg/d)	2.2 ± 0.5	2.2 ± 0.7	0.999	2.0 ± 0.6	2.2 ± 0.8	0.689	0.730	0.999	0.751	0.999	0.609
Niacin (mg/d)	30.4 ± 9.4	31.9 ± 10.5	0.935	29.6 ± 10.4	27.0 ± 10.4	0.532	0.990	0.106	0.706	0.528	0.137
Vit B6 (mg/d)	2.0 ± 0.4	2.9 ± 1.0	**0.001**	2.0 ± 0.5	2.7 ± 1.0	**<0.001**	0.999	0.617	**<0.001**	**0.011**	**<0.001**
Folate (μg/d)	659.8 ± 233.9	715.6 ± 170.1	0.798	666.2 ± 269.2	706.2 ± 243.5	0.794	0.999	0.998	0.742	0.848	0.633
Vit B12 (μg/d)	4.3 ± 2.1	5.6 ± 2.6	0.378	6.4 ± 4.4	5.0 ± 2.4	0.098	**0.035**	0.795	0.658	0.793	**0.027**
Magnesium (mg/d)	509.7 ± 139.8	519.2 ± 129.9	0.995	507.0 ± 163.0	556.7 ± 158.0	0.301	0.999	0.641	0.980	0.569	0.313
Zinc (mg/d)	14.3 ± 4.5	16.3 ± 6.8	0.439	14.3 ± 4.9	15.5 ± 4.3	0.584	0.757	0.254	0.892	0.999	0.225
Selenium (μg/d)	117.8 ± 57.8	131.3	0.655	123.3 ± 43.9	126.9 ± 45.6	0.974	0.957	0.967	0.835	0.836	0.676
Sugar (g/d)	139.4 ± 41.8	129.2 ± 44.5	0.812	138.5 ± 54.5	126.4 ± 38.1	0.477	0.999	0.991	0.752	0.624	0.415
Salt (g/d)	2.6 ± 1.0	1.8 ± 0.5	**<0.001**	2.4 ± 0.8	1.9 ± 0.6	**0.001**	0.674	0.926	**0.001**	**<0.001**	**<0.001**
Fiber (g/d)	44.0 ± 17.0	48.2 ± 20.6	0.792	44.6 ± 16.1	45.6 ± 16.8	0.988	0.999	0.901	0.761	0.980	0.748
Caffeine (mg/d)	165.1 ± 138.1	143.4 ± 106.9	0.820	122.0 ± 76.5	123.7 ± 85.5	0.999	0.252	0.765	0.718	0.284	0.222
Black tea (mL/d)	519.0 ± 463.5	854.4 ± 817.6	0.127	485.7 ± 517.8	666.6 ± 529.5	0.370	0.995	0.425	**0.018**	0.732	**0.021**

nutrients

Supplementary Material

MDPI

[a] Cases with ≤7 score for anxiety (normal); [b] Controls with ≤7 score for anxiety (normal); [c] Cases with >7 score for anxiety (having anxiety); [d] Controls with >7 score for anxiety (having anxiety); [f] One-way ANOVA with Tukey post hoc was used for the comparison. *Significant values are given in **bold**. SFA= saturated fatty acids; MUFA= monounsaturated fatty acids; PUFA= polyunsaturated fatty acids; Vit= vitamin.

Supplementary Table S3. Comparison (mean ± SD) of dietary intakes (macro- and micronutrients) of participants in stress subgroups – here with normal vs. elevated levels of stress [f]*.

**Dietary intakes**	**Cases ≤14 score (n=64)[a]**	**Controls ≤14 score (n=81)[b]**	***p*-value (a *vs.* b)**	**Cases >14 score (n=18)[c]**	**Controls >14 score (n=14)[d]**	***p*-value (c *vs.* d)**	***p*-value (a *vs.* c)**	***p*-value (b *vs.* d)**	***p*-value (b *vs.* c)**	***p*-value (a *vs.* d)**	***p*-value (Between Groups)**
Energy intake (kcal/d)	2996 ± 662.9	3007 ± 542.5	0.999	3070 ± 480.6	2895 ± 596.9	0.836	0.965	0.911	0.977	0.936	0.686
Protein (g/d)	102.5 ± 41.4	112.2 ± 41.3	0.457	96.3 ± 30.7	93.0 ± 24.6	0.995	0.935	0.333	0.410	0.844	0.168
Carbohydrates (g/d)	381.5 ± 119.7	371.4 ± 114.2	0.956	376.3 ± 94.5	385.3 ± 142.5	0.996	0.999	0.975	0.998	0.999	0.950
Fat (g/d)	116.8 ± 42.6	108.1 ± 33.5	0.515	130.0 ± 43.3	97.8 ± 25.4	0.081	0.555	0.782	0.118	0.324	**0.050**
SFA (g/d)	49.8 ± 37.1	39.6 ± 27.4	0.242	65.2 ± 40.6	43.1 ± 22.6	0.222	0.286	0.982	**0.015**	0.899	**0.017**
MUFA (g/d)	29.9 ± 11.1	30.5 ± 9.6	0.981	27.4 ± 12.4	27.9 ± 9.9	0.999	0.814	0.818	0.666	0.918	0.614
PUFA (g/d)	28.0 ± 17.3	32.9 ± 21.1	0.387	28.4 ± 14.1	22.2 ± 14.6	0.787	0.999	0.198	0.789	0.723	0.154
Vit A (μg/d)	594.1 ± 207.4	702.6 ± 384.5	0.164	552.7 ± 189.8	660.5 ± 345.1	0.764	0.957	0.966	0.252	0.890	0.111
Beta-carotene (μg/d)	4557 ± 1908	5884 ± 2260	**0.001**	4711 ± 1482	5054 ± 2610	0.968	0.993	0.523	0.144	0.854	**0.002**
Vit D (μg/d)	2.1 ± 1.6	2.4 ± 1.7	0.498	1.2 ± 1.0	2.2 ± 1.0	0.285	0.138	0.948	**0.011**	0.993	**0.021**
Vit E (mg/d)	17.9 ± 8.0	19.0 ± 6.9	0.822	23.9 ± 11.1	16.3 ± 5.0	**0.034**	**0.022**	0.631	0.078	0.822	**0.020**
Vit C (mg/d)	153.6 ± 70.7	162.2 ± 57.7	0.865	157.2 ± 94.6	149.4 ± 43.1	0.988	0.997	0.908	0.991	0.996	0.839
Thiamin (mg/d)	2.0 ± 0.8	2.2 ± 1.0	0.642	2.0 ± 0.6	2.2 ± 0.9	0.945	0.999	0.999	0.878	0.906	0.637
Riboflavin (mg/d)	2.1 ± 0.6	2.1 ± 0.7	0.999	2.0 ± 0.5	2.5 ± 1.1	0.218	0.929	0.306	0.896	0.285	0.247
Niacin (mg/d)	30.2 ± 9.9	28.8 ± 11.0	0.850	28.4 ± 10.7	29.8 ± 9.0	0.981	0.914	0.988	0.999	0.999	0.840
Vit B6 (mg/d)	2.0 ± 0.5	2.7 ± 1.0	**<0.001**	2.0 ± 0.3	2.8 ± 0.9	**0.041**	0.999	0.999	**0.006**	**0.009**	**<0.001**
Folate (μg/d)	664.2 ± 234.5	687.3 ± 184.3	0.935	661.1 ± 334.4	840.9 ± 324.4	0.140	0.999	0.111	0.973	0.055	0.080
Vit B12 (μg/d)	5.4 ± 2.8	5.2 ± 2.4	0.984	7.1 ± 6.5	5.7 ± 3.3	0.598	0.185	0.951	0.103	0.989	0.148
Magnesium (mg/d)	494.2 ± 149.6	551.6 ± 145.6	0.107	556.2 ± 169.2	485.6 ± 135.5	0.555	0.411	0.432	0.999	0.997	0.078
Zinc (mg/d)	15.0 ± 4.3	15.2 ± 5.3	0.999	15.6 ± 4.7	14.6 ± 7.9	0.946	0.968	0.983	0.986	0.994	0.951
Selenium (μg/d)	116.6 ± 44.4	127.2 ± 42.7	0.492	139.6 ± 57.9	137.3 ± 37.8	0.999	0.219	0.861	0.709	0.398	0.147
Sugar (g/d)	133.8 ± 51.7	124.5 ± 36.9	0.579	156.2 ± 43.7	145.2 ± 40.7	0.897	0.227	0.361	**0.031**	0.814	**0.029**
Salt (g/d)	2.4 ± 0.8	1.8 ± 0.5	**<0.001**	2.7 ± 1.0	2.2 ± 0.5	0.141	0.269	0.241	**<0.001**	0.777	**<0.001**
Fiber (g/d)	45.0 ± 17.1	47.0 ± 19.0	0.902	42.2 ± 12.9	44.5 ± 13.8	0.983	0.934	0.961	0.721	0.999	0.724
Caffeine (mg/d)	140.7 ± 103.6	122.0 ± 85.7	0.650	115.5 ± 88.5	187.1 ± 126.0	0.162	0.761	0.094	0.994	0.363	0.094

Black tea (mL/d)	509.3 ± 511.5	683.4 ± 519.2	0.291	448.1 ± 463.2	1075 ± 1171	**0.016**	0.980	0.100	0.417	**0.007**	0.005

[a] Cases with ≤14 score for the stress (normal); [b] Controls with ≤14 score for the stress (normal); [c] Cases with >14 score for the stress (having stress); [d] Controls with >14 score for the stress (having stress); [f] One-way ANOVA with Tukey post hoc was used for the comparison. *Significant values are given in **bold**. SFA= saturated fatty acids; MUFA= monounsaturated fatty acids; PUFA= polyunsaturated fatty acids; Vit= vitamin.

Supplementary Table S4. Pearson correlation coefficients and *p*-values between dietary intakes (macro- and micronutrients) and scores of depression, anxiety, and stress in cases, controls, and total sample[f]*.

**Dietary intakes**	**Depression**			**Anxiety**			**Stress**		
	**Cases (n=82)**	**Controls (n=95)**	**Total (n=177)**	**Cases (n=82)**	**Controls (n=95)**	**Total (n=177)**	**Cases (n=82)**	**Controls (n=95)**	**Total (n=177)**
Energy intake (kcal/d)	-0.021; 0.849	-0.034; 0.724	-0.029; 0.701	-0.013; 0.907	-0.083; 0.424	-0.047; 0.537	0.015; 0.981	-0.052; 0.617	-0.014; 0.585
Protein (g/d)	0.062; 0.578	-0.007; 0.947	0.034; 0.657	-0.019; 0.864	-0.170; 0.099	-0.109; 0.149	-0.119; 0.288	**-0.267; 0.009**	**-0.212; 0.005**
Carbohydrates (g/d)	-0.052; 0.664	0.086; 0.406	0.020; 0.793	-0.021; 0.853	-0.137; 0.185	-0.082; 0.279	-0.017; 0.878	0.066; 0.523	0.032; 0.666
Fat (g/d)	0.007; 0.954	-0.199; 0.053	-0.100; 0.186	0.020; 0.861	0.158; 0.126	0.096; 0.201	0.095; 0.395	-0.059; 0.569	0.058; 0.444
SFA (g/d)	-0.053; 0.635	-0.035; 0.739	-0.059; 0.432	0.094; 0.403	0.120; 0.248	0.117; 0.121	0.135; 0.228	0.006; 0.951	0.114; 0.131
MUFA (g/d)	0.072; 0.521	-0.138; 0.183	-0.027; 0.720	-0.141; 0.205	0.009; 0.930	-0.069; 0.363	-0.171; 0.125	-0.087; 0.400	-0.136; 0.072
PUFA (g/d)	0.143; 0.199	**-0.210; 0.041**	-0.054; 0.475	-0.089; 0.428	0.095; 0.359	0.013; 0.865	0.074; 0.508	-0.059; 0.568	-0.020; 0.796
Vit A (µg/d)	-0.089; 0.427	-0.067; 0.520	-0.055; 0.475	-0.159; 0.153	**-0.223; 0.030**	**-0.206; 0.006**	-0.023; 0.834	-0.006; 0.952	-0.046; 0.546
Beta-carotene (µg/d)	-0.148; 0.427	0.033; 0.752	-0.018; 0.817	0.015; 0.892	0.170; 0.100	0.081; 0.282	0.076; 0.499	**-0.202; 0.049**	-0.131; 0.082
Vit D (µg/d)	-0.107; 0.339	0.026; 0.802	-0.021; 0.781	-0.193; 0.082	0.081; 0.437	-0.055; 0.468	-0.159; 0.154	0.012; 0.906	-0.098; 0.195
Vit E (mg/d)	0.114; 0.308	-0.144; 0.165	-0.006; 0.933	0.102; 0.364	0.004; 0.966	0.058; 0.444	**-0.352; 0.001**	-0.156; 0.132	0.129; 0.087
Vit C (mg/d)	**0.267; 0.015**	0.030; 0.777	**0.161; 0.030**	0.071; 0.528	0.001; 0.998	0.034; 0.656	-0.006; 0.958	-0.029; 0.777	-0.025; 0.745
Thiamin (mg/d)	-0.048; 0.670	0.096; 0.353	0.045; 0.555	-0.077; 0.494	0.037; 0.724	-0.016; 0.830	-0.046; 0.663	-0.074; 0.475	-0.081; 0.285
Riboflavin (mg/d)	0.130; 0.245	-0.143; 0.168	-0.031; 0.682	-0.145; 0.194	0.096; 0.353	0.000; 0.999	-0.002: 0.982	0.077; 0.461	0.031; 0.685
Niacin (mg/d)	0.183; 0.099	-0.191; 0.063	-0.023; 0.756	0.085; 0.444	**-0.212; 0.039**	-0.076; 0.315	-0.180; 0.105	0.076; 0.463	-0.034; 0.655
Vit B6 (mg/d)	**-0.222; 0.049**	0.071; 0.493	0.018; 0.808	-0.008; 0.941	-0.104; 0.314	-0.098; 0.193	0.025; 0.825	-0.110; 0.290	-0.136; 0.072
Folate (µg/d)	-0.025; 0.827	0.071; 0.496	0.029; 0.698	-0.058; 0.602	0.051; 0.623	-0.012; 0.874	-0.071; 0.258	0.117; 0.258	-0.001; 0.933
Vit B12 (µg/d)	**-0.262; 0.017**	-0.093; 0.369	0.103; 0.171	0.096; 0.392	**-0.253; 0.014**	-0.047; 0.535	0.060; 0.524	0.130; 0.208	0.101; 0.183
Magnesium (mg/d)	-0.036; 0.750	-0.037; 0.721	-0.027; 0.720	0.010; 0.927	0.182; 0.077	0.091; 0.222	0.158; 0.157	-0.152; 0.142	-0.021; 0.777
Zinc (mg/d)	**-0.284; 0.010**	**-0.214; 0.037**	0.012; 0.874	0.111; 0.325	-0.094; 0.367	-0.012; 0.870	-0.005; 0.967	0.081; 0.433	0.046; 0.547
Selenium (µg/d)	-0.118; 0.292	0.093; 0.370	-0.007; 0.925	0.101; 0.362	-0.051; 0.620	0.017; 0.817	0.157; 0.158	0.007; 0.945	0.067; 0.375
Sugar (g/d)	0.034; 0.761	**0.331; 0.001**	**0.157; 0.037**	0.059; 0.599	-0.042; 0.684	0.021; 0.777	0.104; 0.353	0.193; 0.061	**0.164; 0.029**
Salt (g/d)	-0.007; 0.951	0.116; 0.263	0.011; 0.888	-0.041; 0.717	0.103; 0.321	0.053; 0.486	0.135; 0.227	0.135; 0.192	**0.197; 0.009**

nutrients

Supplementary Material

MDPI

Fiber (g/d)	-0.090; 0.422	0.017; 0.872	-0.025; 0.743	-0.004; 0.974	-0.108; 0.297	-0.068; 0.368	-0.024; 0.832	-0.086; 0.408	-0.069; 0.630
Caffeine (mg/d)	-0.040; 0.724	-0.008; 0.942	-0.025; 0.746	-0.198; 0.075	0.004; 0.971	-0.090; 0.234	0.035; 0.758	0.108; 0.298	0.074; 0.328
Black tea (mL/d)	0.077; 0.490	-0.178; 0.085	-0.056; 0.457	-0.161; 0.148	-0.095; 0.362	**-0.148; 0.049**	-0.060; 0.594	**-0.228; 0.026**	0.064; 0.395

[f] Continues variables were used to obtain the Pearson correlation coefficient and p-values. *Significant values are given in **bold**. SFA= saturated fatty acids; MUFA= monounsaturated fatty acids; PUFA= polyunsaturated fatty acids; Vit= vitamin
